# Capgras delusion for animals and inanimate objects in Parkinson’s Disease: a case report

**DOI:** 10.1186/s12888-015-0460-7

**Published:** 2015-04-08

**Authors:** Lucrezia Islam, Sylvie Piacentini, Paola Soliveri, Silvio Scarone, Orsola Gambini

**Affiliations:** 1 University of Milan Medical School, Ospedale San Paolo, Via A.Di Rudini 8, 20142 Milan, Italy; 2 Movement Disorder Department, Istituto Neurologico Carlo Besta, Via Celoria 11, Milan, Italy

**Keywords:** Parkinson’s Disease, Capgras, Dopaminergic psychosis

## Abstract

**Background:**

Capgras delusion is a delusional misidentification syndrome, in which the patient is convinced that someone that is well known to them, usually a close relative, has been replaced by an impostor or double. Although it has been frequently described in psychotic syndromes, including paranoid schizophrenia, over a third of the documented cases of Capgras delusion are observed in patients with organic brain lesions or neurodegenerative disease, including Parkinson’s Disease. Variants of Capgras involving animals or inanimate objects have also been described. The etiology of Capgras in Parkinson’s remains unclear, but may arise from a combination of factors, such as frontal lobe dysfunction and dopaminergic medication.

**Case presentation:**

We present the case of a 53-year old right-handed female with Parkinson’s disease who developed Capgras delusion during treatment with dopamine agonists and Levodopa/Carbidopa. She became convinced that her pet dogs and the plants in her garden had been substituted by identically looking ones. Our patient was initially treated with Quetiapine, with no improvement, and subsequently treated with Clozapine, which lead to partial regression of her symptoms. Neuropsychological Evaluation showed Mild Cognitive Impairment in Executive Functions.

**Conclusions:**

Given the clinical history, onset and evolution of symptoms we believe our patient’s delusion resulted from the overlap of dopaminergic medication and Mild Cognitive Impairment in executive functions. Zoocentric Capgras, the variant we describe, has been rarely described in scientific literature, and we believe it is of interest due to its unusual characteristics.

## Background

Capgras delusion is a delusional misidentification syndrome, in which patients become convinced that someone close to them has been replaced by an impostor. Joseph Capgras first described this delusion in 1923, and called it “l’illusion des sosies”. Capgras and Reboul-Lachaux described the case of a woman who, among other psychotic symptoms, developed the delusional belief that her husband, children, neighbors, and others had been replaced by doubles [1,2]. Capgras syndrome involving animals or inanimate objects has been rarely described in scientific literature [3-5].

Although it has been frequently described in psychotic syndromes, including paranoid schizophrenia [6-9], over a third of the documented cases of Capgras syndrome occur in patients with organic brain lesions, suggesting that the syndrome may have an organic etiology [8,10,11]. Capgras may also be observed in Parkinson’s Disease (PD) [12,13], and may arise as a consequence of dopaminergic medication and frontal lobe dysfunction.

## Case presentation

Our patients is a 53-year old right-handed Caucasian female, who has been married for over 20 years, has a 20 year old son, and works with her husband in a family run business. She initially presented with tremor and stiffness, predominantly on her right side, in 2011, for which her GP referred her to Istituto Neurologico Besta. Clinical history was unremarkable, and there was no family history of psychiatric or movement disorders. She underwent a DAT-scan in September 2012, which revealed presynaptic dopaminergic deficit. At the time, her MMSE score was 29/30; no affective or delusional symptoms were noted at the outpatient visits or were reported by family members. Brain MRI was normal. She was diagnosed with PD, and in March 2013 she was started on extended release Pramipexole up to 2.1 mgs, with moderate improvement in her motor symptoms. The medication choice was dictated by the evidence of lower risk in motor complications compared to levodopa, in keeping with current treatment guidelines [14]. In July 2013, Levodopa/Carbidopa at the dose of 100/25 mg 3 times daily was added to her medication, and led to significant improvement of motor symptoms. In the summer of 2013 her husband noticed hyperactivity and a dramatic increase in her hobby activity: aside from excessive goal directed activity, he did not report other symptoms such as mood elevation, dysphoria, reduced need for sleep or grandiosity; the patient’s hyperactivity regressed after Pramipexole was withdrawn (whereas Levodopa/Carbidopa was maintained). Towards the end of November 2013 she started experiencing delusional symptoms: one day she noticed something strange in the behavior of some of the handy-men she had hired to do some work around the house. She also became convinced that some of the paintings in her house had been replaced, and that the frames also seemed different. In the following days she noticed that her dogs had also been replaced: the impostors were almost identical to the original dogs but they had a slightly different color and their spots were in different places. She became convinced that the cypresses in her garden had been replaced as well, and she was quite sure about this because the new trees were younger and smaller than the original ones. When asked who was responsible for replacing them or what may have lead them to do such a thing she stated that she herself was puzzled about this, and that she found all these events very bizarre. She was also unable to pinpoint the exact differences between the impostors and the original objects and pets. When asked why she thought her husband or others around her could not notice these differences she said she was not sure, but she thought this might be explained by the fact that they only superficially observed the objects, plants and animals. Interestingly, her delusion never involved her family members or other people close to her. In October, a psychiatric consultation was requested: mood and affect were normal, her *Structured Clinical Interview for DSM-IV Axis I Disorders* (SCID-I) did not reveal any previous symptoms consistent with a diagnosis of lifetime or current affective, anxiety, substance abuse, eating or somatoform disorder. Her *Brief Psychiatric Rating Scale* (BPRS) score at the time was 38, indicating a ‘moderatly ill’ patient. The patient symptoms were consistent with a diagnosis of current delusional disorder, for which she started on a low dose of Quetiapine (50 mg) with no significant improvement in psychiatric symptoms: she was very worried and anxious, and she rapidly became irritable when she was contradicted by either her husband or her doctors. The choice of Quetiapine over other medication was dictated by a relatively favorable motor side effect profile compared to other anitpyshcotics; the choice of Quetiapine over Clozapine as a first line medication was motivated by the possbile side effects of Clozapine (particularly agranulocitosis) and the fact that Clozapine therapy requires weekly monitoring of blood panel for 18 weeks.

The patient did not undergo any further brain imaging. In April 2014 the patient was started on low dose Clozapine, starting with 12.5 mgs, and slowly increased to 50 mgs, and her symptoms partially regressed. The dose of Clozapine could not be increased further due to sedation. At her last evaluation in November 2014 she was euthymic, had gained insight about her delusion, and had good social and work functioning. The delusions had not completely disappeared, and she still sometimes had the feeling that some items might have been replaced. However, the degree of conviction about the delusions had significantly decreased, and her and anxiety has improved dramatically. BPRS score was 23.

### Neuropsychological assessment

The patient underwent neuropsychological evaluation in September 2014 (Table 1). Her Montreal Cognitive Assessment (MoCA) was impaired. Memory, praxia, face recognition, visual object and space perception were normal. The patient was slighty impaired in graphic fluency and had a few slips of attention. There was no evidence of functional impairment. Global assessment fulfilled diagnostic criteria for Mild Cognitive Impairment (MCI) in PD [15] single domain (executive functions).

**Table 1:** **Neuropsychological assessment**

	Raw score*	Adjusted score or cut-off	Equivalent score or classification
***Montreal Cognitive Assessment***	21/30	17.16	0
***Memory***			
*Verbal memory*			
Digit span (forward)	6	5.75	3
Rey 15 word test			
Learning	38/75	35.7	2
Recall	6/15	5.40	1
Recognition	12/15		Average
*Visual memory*			
Visuo-spatial span (forward)	5	4.74	2
Rey-Osterrieth Complex figure Test (RCFT) Recall	12.5/36	13.55	3
***Attention and executive functions***			
Digit span (backward)	4	3.71	2
Visuo-spatial span (backward)	5	4.67	4
Multiple Features Target Cancellation (MFTC)			
Time	37	≤135.73	Average
Correct items	12	≥8.53	Average
False alarm	0	≤2.77	Average
Accuracy	0.96	≥0.869	Average
Stroop color naming			
Time interference	18	17.75	4
Error interference	1	1.05	3
Trail Making Test			
Part A	39	33.60	4
Part B	80	66	4
B-A	41	32.40	4
Phonemic fluency	25	21.90	1
Cognitive Estimation Test (CET)			
Errors	18	≤19	Average
“Bizarre” errors	5	≤5	Borderline
Modified Five Point Test			
Drawings	16	≥23.84	Impaired
Number of strategies	2	3.39	2
***Language and perception***			
Semantic fluency	49	46.90	4
Famous face naming test	78/78	≥53	Average
Benton facial recognition test	47/54	47	Average
Clock drawing	9/10	≥7	Average
Rey-Osterrieth Complex figure Test (**RCFT**) Copy	34/36	33.65	4
Visual Object and Space Perception Battery **(V.O.S.P.)**			
Object perception			
Screening	20/20	≥15	Average
Incomplete letters	19/20	≥16	Average
Silhouettes	26/30	≥15	Average
Object identification	19/20	≥14	Average
Progressive silhouettes	8/20	≤15	Average
Spatial perception			
Counting	10/10	≥8	Average
Position discrimination	20/20	≥18	Average
Number localization	7/10	≥7	Borderline
Cube analysis	10/10	≥6	Average

*Legend: *Raw score*: score test; *Adjusted score*: obtained by adding or subtracting the contribution of patient’s age and education; *Equivalent score*: adjusted scores converted to a five-point interval scale, from 0 to 4 equivalent scores. The five- point interval scale is divided as follows: 0 = scores equal or lower than the outer tolerance limit (5%); 4 = scores higher than the median value of the whole sample; 1, 2 and 3 are obtained by dividing into three equal parts the area of distribution between 0 and 4 [16].

All the procedures described in the above sections are in compliance with the Declaration of Helsinski and were approved by the Ethics Committee at Istituto Neurologico Carlo Besta.

## Conclusions

Capgras is a relatively rare delusion, and is mostly described in patients with concomitant brain lesions or neurodegenerative disease, including PD [11,12,17]. Typical Capgras delusion involves a close relative, thought there have been some reports of Capgras involving animals [5] and inanimate objects [3,4].

Face perception is a function with significant complexity, which is reflected in hierarchical cognitive models. Current neuroimaging data show that face perception involves a core-processing network of cortical modules, each of which is specialized in different functions involved in face processing [18-20]. Overall it is a highly specialized network, and differs from other visual information processing [21-23]. Capgras delusion can be considered a disorder of face processing, and some studies have proposed that Capgras may be a mirror image of prosopoagnosia [24], which is characterized by impaired face recognition, usually arising as a result of bilateral lesions in the inferior temporal lobes [18,25,26]. Contrary to prosopoagnosia, however, patients suffering from Capgras delusion are not impaired in overt face recognition, which is mediated by the ventral route from the visual centers to the temporal lobes, but the dorsal visual route, which is crucial for attribution of emotional significance to a face, is damaged [24]. Physiological studies in patients with Capgras delusion have shown impaired autonomic arousal to familiar faces, which may underpin the belief that others have been replaced by impostors [27-29].

These neurobiological mechanisms, however, only explain the absence of emotional significance which occurs while viewing a familiar face; they don’t shed any light on the absence of cognitive reappraisal of the delusional idea that loved ones have been replaced by doubles. The visual recognition of a familiar face results from both the conscious recognition of the face and the limbic-mediated emotional arousal that accompanies the conscious recognition, which is responsible for the sense of familiarity [29]. Patients with Capgras delusion do not only lack familiarity with their loved ones, they also explain this unusual sensation with a delusion. Ramachandran argues that Capgras may arise from the *conjunction* of two lesions: one affecting the brain’s ability to attach emotional significance to a familiar face, and one affecting the global consistency-checking mechanism in the right hemisphere [28]. A recent imaging study has proposed that Capgras may result from dysfunctional neural activity in parts of the left extended face processing system, which are also activated in Theory of Mind tasks, and abnormalities in the right middle frontal gyrus [30].

Psychiatric symptoms in PD are very often related to cognitive decline [31,32], but the exact patho-physiological mechanisms underpinning of Capgras in PD remain unclear. It has been proposed that delusional misidentification in PD may result from combination of dopaminergic psychosis and cognitive decline [33,34], excessive dopamine associated with *L*-dopa administration, diminished acetylcholine, or both [17]. Here we describe a variant of Capgras involving our patient’s domestic pets and inanimate objects, but sparing her husband and son. The neurobiological underpinnings of variants of Capgras delusion not involving the visual processing of human faces remain unclear, but may result from decoupling of visual recognition of objects and attribution of significance. Similarly to “regular” Capgras, the patients are not impaired in overt object recognition, but fail to identify the object as their own (i.e. lack of familiarity). Moreover, there is a cognitive bias which results in the delusional explanation of said lack of familiarity (“it doesn’t look familiar, therefore it must be a double”).

Given the clinical history, onset and evolution of symptoms we believe our patient’s delusion resulted from the overlap of dopaminergic medication and MCI in executive functions.

Clinicians should be aware of the possibility of misidentification syndromes, including Capgras delusion, in patients with PD, particularly in those with MCI receiving dopaminergic medication.

### Consent

The patient was provided with a thorough explanation of all procedures before she signed informed consent. Informed consent was obtained for publication of this case report. A copy of the written consent is available for review by the Editor of this journal.

## Abbreviations

PD: Parkinson’s Disease
SCID-I: Structured Clinical Interview for DSM-IV Axis I Disorders
BPRS: Brief Psychiatric Rating Scale
MoCA: Montreal Cognitive Assessment
MCI: Mild Cognitive Impairment
